# Ambient Sulphur Dioxide and Female ED Visits for Migraine

**DOI:** 10.5402/2012/279051

**Published:** 2012-03-15

**Authors:** Mieczysław Szyszkowicz, Eugeniusz Porada

**Affiliations:** Population Studies Division, Health Canada, 269 Laurier Avenue, Room 3-030, Ottawa, ON, Canada K1A 0K9

## Abstract

Ambient sulphur dioxide (SO_2_) concentrations may affect the number of female emergency department (ED) visits for migraine. ED visits diagnosed as migraine among females in two cities in Canada, Toronto (*N* = 704) and Ottawa (*N* = 3, 358), were analyzed. In the study case-crossover design was used. Conditional logistic regression was realized to estimate odds ratios (ORs) and their 95% confidence intervals (CIs) relative to an increase in an interquartile range (IQR, in Toronto IQR = 2.9 ppb, in Ottawa IQR = 3.9 ppb) of sulphur dioxide. In the constructed conditional logistic regression models, temperature and relative humidity were adjusted in the form of natural splines. In Toronto positive and statistically significant associations of sulphur dioxide with migraine ED visits were obtained: all ages, OR = 1.04 (95% CI: 1.00, 1.08); age group [15, 50], OR = 1.05 (95% CI: 1.01, 1.09). In Ottawa positive correlations were observed: all ages, OR = 1.05 (95% CI: 0.97, 1.13); age group [15, 50], OR = 1.06 (95% CI: 0.97, 1.15). The results suggest that female migraine may be affected by ambient sulphur dioxide.

## 1. Introduction

The aim of this paper was to investigate potential correlations between ambient sulphur dioxide and female emergency department (ED) visits for migraine in two Canadian cities, Toronto and Ottawa, two locations with different levels of the pollutant concentrations. Case-crossover (CC) design was used [1]. Conditional logistic regression was applied to realize calculations (PHREG procedure, SAS, v. 9.1) related to the CC technique. Daily events, diagnosed on ED as migraine, were used to represent health outcomes. The considered sulphur dioxide concentrations, temperature (dry bulb), and relative humidity were expressed as daily mean values.

This study was inspired by a few recent publications [2–5], related to investigation ambient air pollution exposure and ED visits for migraine and headache. The published results suggested the associations of ambient sulphur dioxide concentrations with female ED visits for migraine. 

## 2. Materials and Methods

### 2.1. Study Population

Population considered in this study were patients served by two different hospitals in Canada: in Toronto and Ottawa. In Toronto we considered the period between April 1, 1999 and March 31, 2002. In this time period we had 898 ED visits diagnosed as migraine, where 704 cases (78%) were females. In Ottawa we considered the period between April 1, 1992 and December 31, 2000, and we identified 4,568 migraine cases, 74% among them (3,358 cases) were female patients. We considered patients of all ages. We applied the ICD-9 codes [6] to identify the cases classified as migraine and we used 346.X rubric in both cities. In our calculations, we considered all patients. The presented graphical figures were produced for the patients of age in the interval [0, 85] years. Thus in this situation we put an age restriction.

### 2.2. Statistical Analysis

The CC technique is an adaptation of the case-control study [1]. By definition of the case-crossover methodology, the cases acted as their own controls on a set of predefined control days proximate to the time they became cases. A time-stratified approach to determine controls was widely accepted and adopted as it has been shown to produce unbiased conditional logistic regression estimates [7]. In the design, the controls are matched to case periods by day of week for the case period (day), and the control periods are determined as other days in the same month and year (controls ± 7 days). This strategy was used here, thus 3 or 4 controls are present for each case. For example, for the case on Sunday, January 1, 2012, other Sundays in January 2012 will be control periods (January 8, 15, 22, and 29). In this situation, we have 4 controls.

In addition, to analyse less stable ED data in Ottawa (i.e., more cases but lower levels of ambient sulphur dioxide than in Toronto), two other strategies to define control periods for the CC method were used [8, 9]. One approach defines control days for a particular case to be every third day within the same month and year that ED visit occurred (control ± 3 days). The second proposed technique uses all days in the same month but 6 days around the case day (all days in a month without 13 days). In this situation, we may have 18, 17, 16, or 15 (February with 28 days) control days (control [−6, 6]) for each case-day. For these sets of time control periods day of week was included in the conditional logistic regression model as an indicator variable.

The generated results were presented as the odds ratios (ORs) and their 95% confidence intervals (95% CIs). The calculated ORs were reported for an increase in the concentration represented as an interquartile range (IQR = Q3–Q1 = 75th–25th values of percentiles, IQR = 2.9 and 3.9 ppb in Toronto and Ottawa, resp.) of the sulphur dioxide level. Temperature and relative humidity were used in form of natural splines with 3 degrees of freedom. All components of the models, sulphur dioxide, and meteorological factors in the models were represented by values on the same day as the visit day. Here we only analyzed the same day exposure.

In addition, the following calculations were executed. The sequence of 57 overlapping age groups, each of length 30 years, was defined in the following way: the first age group was [0, 29], second [1, 30], and each next was created from the current one by shifting it by one year. In this sequence, the last element was defined to be the age group [56, 85]. The length of the age groups (here 30 years) was chosen rather arbitrarily but in relation to the number of ED visits. For the elements of this sequence, the CC analysis was performed separately. Thus the first OR corresponds to the age group [0, 29], second [1, 30], and so on. The calculations were executed for female patients only.

### 2.3. Air Pollution Data

Hourly air pollution data were obtained from fixed monitoring stations in the considered cities. These data were supplied by Environment Canada. For each air pollution variable, we have 24 measurements recorded at hourly intervals. The daily shared exposures of the population were expressed as mean values among stations in city (Toronto: 4.6, 2.7, 4.0, and 2.9 ppb; Ottawa: 3.9, 3.1, 3.5, and 3.9 ppb; mean, standard deviation, median, and IQR, resp.)

## 3. Results and Discussion

In Toronto positive and statistically significant associations of sulphur dioxide with migraine were obtained: all ages, OR = 1.04 (95% CI: 1.00, 1.08); age group [15, 50], OR = 1.05 (95% CI: 1.01, 1.09). In Ottawa positive associations (no significant) were observed: all ages, OR = 1.05 (95% CI: 0.97, 1.13); age group [15, 50], OR = 1.06 (95% CI: 0.97, 1.15). Our hypothesis is that ambient sulphur dioxide is associated with ED visits for migraine. The above reported results were obtained using the time-stratified (classical, controls ± 7 days) case-crossover technique. It is possible that by increasing the number of control periods, we may improve our estimations [8].

**Figure 1:**
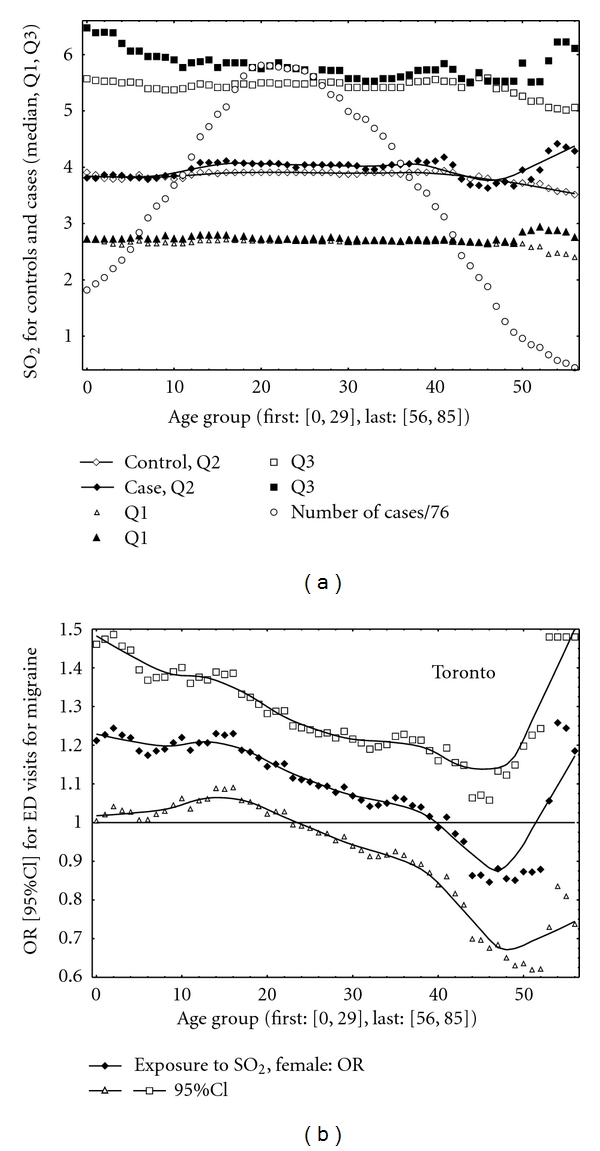
Toronto. (a) Median (Q2), Q1, Q3, cases scaled by 76. (b) ORs with their 95% CIs.

**Figure 2:**
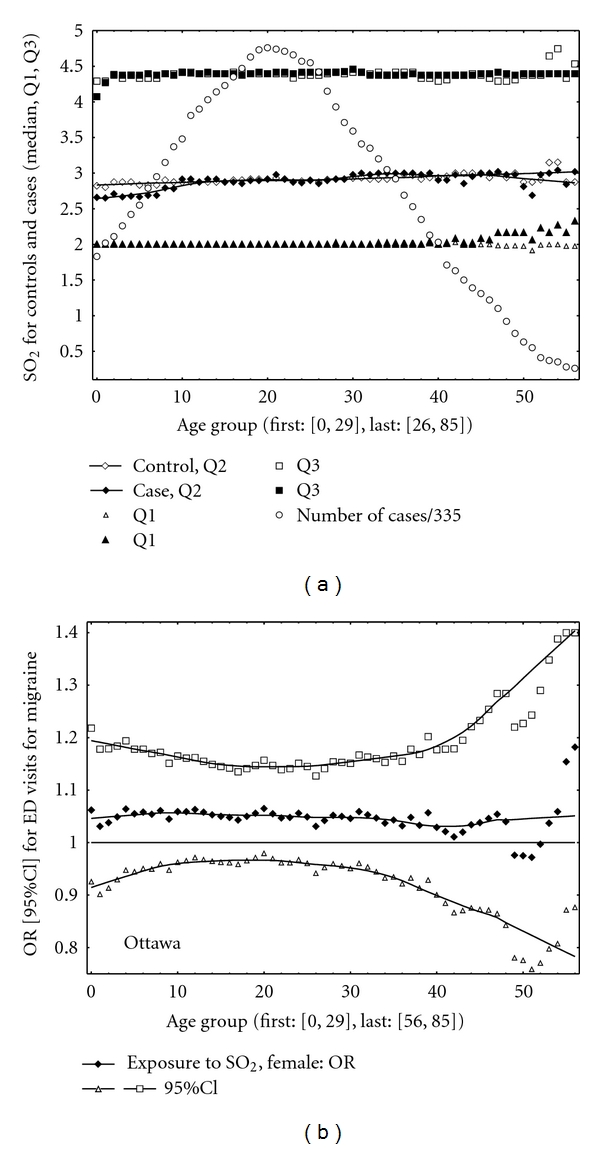
Ottawa. (a) Median (Q2), Q1, Q3, cases scaled by 355. (b) ORs with their 95% CIs.

**Figure 3:**
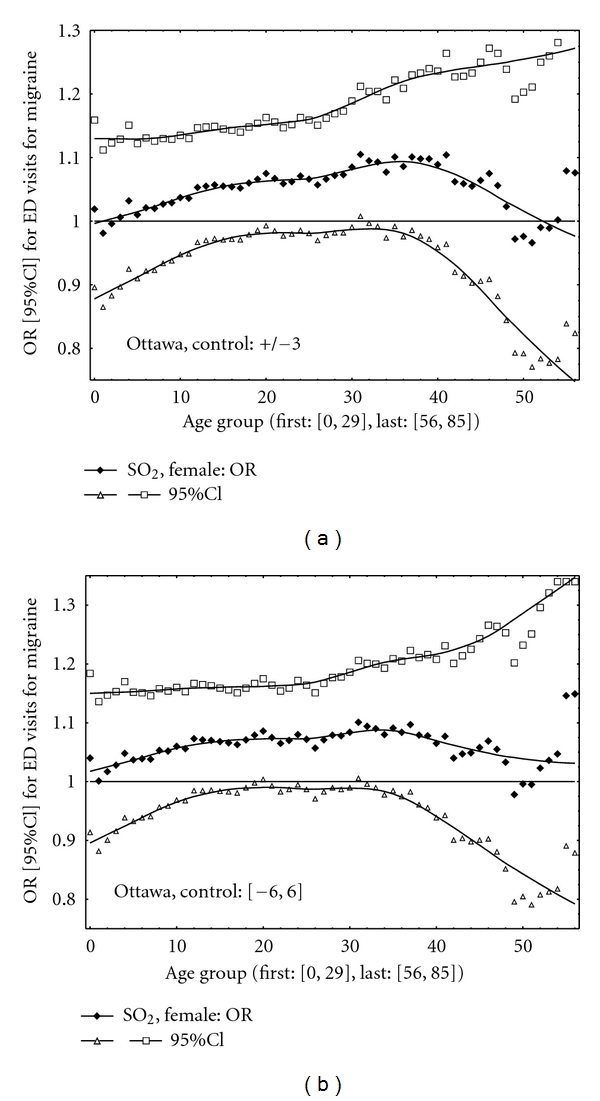
Ottawa. ORs with their 95% CIs. (a) Controls ± 3 days. (b) Controls [−6, 6].

The main results are organized in the form of three figures (Figure 1 for Toronto, Figures 2 and 3 for Ottawa). Two figures, Figures 1 and 2, are composed of two different parts: (a) related to air pollution and (b) related to odds ratios. The top part shows the sulphur dioxide characteristics for controls and cases (the estimated median (Q2), Q1, and Q3) and also indicates the number of cases (divided by 76 and 355, in Toronto and Ottawa, resp.). The numbers of cases were scaled to have values on the same graph with sulphur dioxide characteristics. The bottom part illustrates the values of ORs and the corresponding 95% CIs. The results are shown (and were calculated) for each age group separately.

In Toronto, for many age groups the results were positive and statistically significant (the lower 95% CI limits greater than one, Figure 1). The positive correlations were obtained between the pollutant and migraine in Ottawa (Figure 2). Also should be noted that level of sulphur dioxide (expressed as a median value) was higher in Toronto than that in Ottawa.  Figure 3 shows the results for Ottawa for two different variants of the CC design implementations. For example, the following results were obtained in Ottawa for the CC method with control periods as all days in a month without 13 days ([−6, 6]): age group [15, 50], OR = 1.07 (95% CI: 1.00, 1.16); age group [25, 50], OR = 1.09 (95% CI: 1.00, 1.19); age group [25, 60], OR = 1.08 (95% CI: 1.00, 1.18). Thus the results are close to be statistically significant (*P* value < 0.05).

In this study, statistically significant (in Toronto) short-term effects were observed for ambient sulphur dioxide exposures on daily ED visits for migraine for females. Ambient air pollutant affects patients of different age ranges. The results suggest that above a specific level of sulphur dioxide concentrations or acute air pollution events (large Q3) might result in elevated number of ED visits for this health condition. The shape of the relations by age group (Figures 1 and 2) in Toronto and Ottawa is different. We identified only 704 cases in Toronto versus 3,358 in Ottawa, but Toronto has higher level of ambient sulphur dioxide than Ottawa. Figure 1 may indicate that in Toronto, specific age group is affected by exposure. In Ottawa (Figure 2), the results may suggest that more biological aspects are involved and dominate; the associations are stronger for the age groups where we observe pick of the frequencies of ED visits.

A few published studies have examined the effect of air pollution on migraine. In previous publication related to ED in Ottawa [10], the authors used the same data and did not find correlations between air pollution and ED visits for migraine (see Figure 2). Probably for the same reason, lower level of exposures and consequently weaker contrast between case and control periods, the authors of the work [11] in USA did not note significant correlations in their study. In both mentioned publications, the results for sulphur dioxide were only positive, and in these two papers, the classical time-stratified CC technique was realized (i.e., 3 or 4 control days). In contrast, the study performed on hospital admission for migraine in Chile reported statistically significant associations between air pollution exposures and hospitalization for headache [5]. The authors estimated relative risk for migraine as 1.10 (95% CI: 1.04, 1.17) for one IQR = 6.20 ppb increase in sulfur dioxide. The animal study on Saharan dust has revealed an unknown environmental factor as a possible trigger for headache [12]. In the epidemiological study performed at the New York University, headaches were observed among persons exposed to the World Trade Center dust clouds after 9/11 [13]. Environmental factors including noise, light/sun, heat, cold, fumes, odor, smoke, and vibration were studied in relation to headache among military persons [14]. The authors of [15] reviewed the literature addressing indoor and outdoor environmental factors which are commonly implicated as migraine triggers. In their conclusion they stated: “There are conflicting studies supporting the validity of patient-reported environmental migraine triggers”. In another migraine-related study, headache triggers were questioned in 190 patients. As a result the following factors reported in the study were stressor factors: noise, sleep disturbances, fatigue, hunger, physical effort, light, sun, cold, factors affecting scalp, traveling by motor vehicle, eye strain, crowd, odours, crying, weather, high blood pressure, cigarette smoke, and menstruation, respectively [16].

The limitations of this study are typical of this type of research. They include the adequacy of the model and impact of measurement error in the exposure and outcome variables [8]. The main goal of this work was to investigate female ED visits for migraine in Ottawa. As some other studies indicated the association of ED visits for migraine with exposure to sulphur dioxide, the question was if we have large number of visits (3,358 cases in Ottawa) and relatively low exposure, is it possible to observe associations? We applied the case-crossover methodology with extended number of control periods; this allowed better contrasting exposure in hazard and control periods.

## 4. Conclusions

The results support the hypothesis that ED visits for migraine condition are associated with ambient sulphur dioxide. The large majority of this air pollution arises from combustion of fossil fuels (e.g., diesel motor vehicles).
